# What's Love Got to Do With It? Reflections on the Role of Stuttering in Enabling and Enhancing Relationships

**DOI:** 10.1111/1460-6984.70082

**Published:** 2025-07-03

**Authors:** Amy Connery, Christopher D. Constantino

**Affiliations:** ^1^ Department of Clinical Speech & Language Studies Trinity College Dublin, Ireland; ^2^ School of Communication Science & Disorders Florida State University Tallahassee Florida USA

**Keywords:** clinical implications, love, relationships, stuttering, vulnerability

## Abstract

**Background:**

Despite the literature highlighting the mostly negative impact of stuttering on an individual's life, there is emerging evidence alluding to an alternative and more advantageous experience for some adults who stutter. Features of this alternative experience include enhanced interpersonal relationships and increased sensitivity to others. Investigation of such favourable by‐products of stuttering is lacking in the literature, and in order to comprehensively understand the lived experience of stuttering for all individuals, such exploration is required.

**Aims:**

This article aims to theoretically explore stuttering's capacity to enhance a person's cultivation of loving relationships, and relationships more generally, with others.

**Methods and Procedures:**

This aim is achieved through a broad discussion on the meaning of *love*, and, more specifically, through the examination of the concept of *vulnerability* as a fundamental component that underpins a robust loving relationship. The role of stuttering as an act of vulnerability that has the capacity to enhance the relationships experienced by people who stutter is proposed.

**Main Contribution:**

This paper serves as a novel conversation on the potential for stuttering to enhance a person's cultivation of robust relationships. It continues the discourse that challenges traditional deficit‐based perspectives of stuttering and presents an alternative narrative of stuttering that can shape our research and clinical practices.

**Conclusions and Implications:**

The advantageous by‐products of stuttering, such as the role that stuttering plays in enabling and enhancing relationships, require further exploration. A range of clinical recommendations is outlined in order to support clients’ enactment of vulnerability and enhancement of their relationship experiences.

**WHAT THIS PAPER ADDS:**

*What is already known on the subject*

- The research‐based literature highlights the mostly negative impact that stuttering has on an individual's life. More recent evidence, however, indicates an alternative experience for some adults who stutter, with advantageous by‐products of stuttering such as enhanced interpersonal relationships being reported.

*What this paper adds to existing knowledge*

- This paper discusses the potential for stuttering to enhance a person's cultivation of robust relationships. Early and contemporary theories of love are discussed, and one fundamental component of love, vulnerability, is examined. Stuttering is proposed as being as an act of vulnerability, thus enabling and enhancing relationships.

*What are the potential or actual clinical implications of this work?*

- Speech and language therapists (SLTs) are recommended to support clients to self‐disclose, offer therapies that aim for authenticity and the reduction of concealment, and encourage clients to engage with stuttering support groups.

1

 

We begin to love, rather than merely admire, when others no longer have to exhibit perfection to seem worthy. *de Botton* *2023*, *111*

## 2 Introduction

Our understanding of stuttering and our role as speech and language therapists (SLTs) in supporting people who stutter is continuously evolving. In recent times, conceptualisations of disability such as the social model and the neurodiversity paradigm are increasing in dominance in the literature, and are translating into practice, education, and advocacy work with those who stutter (Bailey et al. 2015; Constantino 2018; Constantino et al. 2022). Pathologising stuttering is increasingly challenged, with some researchers, SLTs and adults who stutter advocating for something beyond merely accepting one's stutter, but rather embracing it with pride and even love (Bailey 2019; Constantino 2019; St Pierre 2019). Literature on the lived experience of stuttering highlights the mostly negative impact that stuttering has on the lives of adults who stutter (Connery et al. 2019). However, on closer examination of this literature, there is evidence that alternative, and more positive, experiences with stuttering are possible for some adults who stutter. Aspects of these experiences include increased sensitivity to others, enhanced interpersonal relationships, and the development of positive self‐identity when supported and included in society (Boyle et al. 2019; Connery et al. 2019). Investigation of such advantageous by‐products of stuttering is lacking in the field, and if we are to comprehensively understand the lived experience of stuttering for all individuals, such exploration is required.

Examination of the psychology literature reveals evidence that experiences perceived by individuals to be negative, such as a traumatic event or a personal loss, can result in positive consequences, termed *post‐traumatic growth* or *adversarial growth* (Jayawickreme and Blackie 2014; Linley and Joseph 2004; Mangelsdorf et al. 2019). Such growth or positive transformation can be manifested as enhanced relationships with others, recognition of new life possibilities, and a heightened appreciation for life (Linley and Joseph 2004; Tedeschi and Calhoun 1996). These findings were corroborated in the stuttering literature, with a mixed methods study by Boyle et al. (2019), who explored the perceived benefits (which the authors termed *benefit finding*) of stuttering and of being a person who stutters. Adults who stutter reported a range of benefits including personal benefits (e.g., emotional growth), relational benefits (e.g., increased sensitivity to others, and a deepening of relationships), and gained perspective (e.g., gained perspective about life and human nature generally, and a sense of appreciation (of communication for example). One specific subtheme of the *Relational Benefits* theme centred on the idea that stuttering can lead to a deeper bond or connection with others, beyond a mere superficial interaction, due to an individual's sharing of personal and challenging stories and experiences relating to their stuttering. Although uncommon in the stuttering literature, this evidence of the more positive consequences of stuttering challenges our intuitive belief that stuttering is solely an adverse life experience. Importantly, the authors of this paper acknowledge that, for many, stuttering *is* a very challenging condition to live with. However, we believe that the alternative or more optimistic perspective on stuttering that some individuals do experience requires increased attention in the literature. Like most human conditions, stuttering is nuanced and complex, containing both negative and positive experiences.

As evidenced in Boyle et al. (2019) study, enhanced relationships with others, resulting from the sharing of stuttering experiences, is a benefit experienced by some adults who stutter. Constantino (2019) discussed the power of stuttering openly, as it facilitates the sharing of one's true self with another and opens up the opportunity for the other person to reveal their true self. Constantino (2019) furthers that this mutual revealing or *vulnerability* cultivates an intimacy and a love of one another. *Love* and *stuttering* are two terms that are rarely, if ever, seen paired in the research‐based literature (cf. Constantino 2025) However, examination of other forms of evidence such as *‘patient‐based evidence*’ (i.e., knowledge generated from the experiences of people who stutter (McCurtin et al. 2019, 377), an often under‐valued form of evidence, demonstrates associations between love and stuttering (Connery et al. 2020; Connery et al. 2021). These *associations* that are evident in books written by people who stutter, relate to a love of talking about one's stuttering, a love for the stuttering community that one has become part of as a result of their stuttering, and also, as previously mentioned, the potential for stuttering to enhance one's ability to develop a loving relationship with another person (Bradberry 2016; Constantino 2019; Reitzes and Reitzes 2012). This article aims to specifically explore the nature of stuttering's capacity to enhance a person's cultivation of loving relationships with others. In order to achieve this aim, it is first necessary to explore the meaning of *love* and examine the definitions of love that have been proposed over the years and across a range of disciplines.

## 3 Defining Love

*Love* is a word we frequently use, but few of us could define it with satisfying accuracy. Therefore, examination of the philosophical and theological literature is helpful. We might start by distinguishing between *love* and similar, analogous concepts. *Love* is clearly more than fondness for something or someone; *loving* is different from *liking*. *Liking* is always transactional, we *like* an object for its instrumental value (what it can do for us); whereas typically we talk of *loving* something or someone for their intrinsic value (what or who they are) (Brown 1987; Singer 1991). Unlike *liking*, *loving* is always relational, because *love* involves identifying ourselves with another (Nussbaum 1990). To *love* you, then, puts me in a relationship with you. I become your friend, wife, father or sister. *Love* must also always be interested in the good of the beloved; it cannot be inherently self‐interested (Whiting 2013). When the beloved suffers, the lover cannot help but suffer; when the beloved experiences joy, the lover cannot help but experience joy as well (Helm 2010). The expansive use of the word *love* in the English language makes the task of definition more difficult. *Love* is used to describe a fondness for a thing or an activity, (e.g., “I love peanut butter and jelly”) or a role that is important to our identity (e.g., “I love being a father”) or to indicate that we care for another as the person they are, (e.g., “I love my wife”) (Helm 2021). It is this last, personal kind of *love* which we are interested in here.

Within personal *love*, there is a traditional Greek distinction amongst *eros*, *philia*, and *agape* (see Plato's Symposium). *Eros* is often called *love of desire*, and theorists typically see it as sexual and selfish (Nygren 1953). *Eros* is neither planned nor willed, rather, it seems to impose itself on the lover (Benedict XVI 2005). The lover is moved by the qualities of the beloved and responds to these qualities (Soble 1989). It is frequently presented as an inferior love compared with *agape* or *philia* because the beloved is not valued in themselves but in response to their transient qualities. For example, if one were to love their partner in an *erosic* sense, they would not love them for being who they are, instead, they might love them in response to their beauty, making the love conditional (Soble 1990). If the beloved were to cease to be beautiful, the lover would cease to love them.

*Philia* is similar to *eros* in that it is typically understood as affection towards others in response to their good qualities (Cooper 1977). However, unlike *eros*, *philia* is concerned with the mutual benefit of the lover and the beloved and is, therefore, not fundamentally selfish (see Aristotle's Nicomachean Ethics, Book VIII). The contemporary notion of *friendship* captures the idea of *philia* quite well (Moseley 2024). True friendship is not transactional, nor is it based only on the pleasure derived from the other's company; rather, it takes pleasure in who the other is in themselves.

The third kind of *love*, *agape*, is true unconditional love, that is, a love that does not respond to the value of its object or the beloved's fundamental characteristics (Badhwar 2003). Through the Christian tradition, *agape* has come to represent both the love God has for us persons, as well as the love persons have for God and their brotherly love for all mankind (Helm 2021). With *agape*, the lover's love is ‘spontaneous and unmotivated’ (Nygren 1953, 85). The lover does not love the beloved because their qualities make them valuable, as in *eros* and *philia*. Rather, the lover loves the beloved first and unconditionally—the love precedes and creates the value. A child is not valuable to a parent because of the child's characteristics; the parent loves the child regardless of its qualities. We might think of the love between two spouses as beginning as a combination of *eros and philia* but evolving into *agape*. Speaking of *agape*, the philosopher Thomas Aquinas said that to *love* is to consistently will and choose the good of the other (Aquinas 1948). For Aquinas, *love* is a choice, not a feeling or imposition.

This tripartite Greek distinction is useful for coming to a single conception of the English word *love*. For the sake of this article and our discussion herein, we will present *love* as a single reality and concept. Plainly, *love* captures the parts of *eros* that suggest that it is a felt experience rather than an act of the will, there is something of infatuation in *love*. As the lover draws near to the beloved, they become less and less concerned with themselves and increasingly seek the good of the other. A mutual fondness or *philia* develops, as well as a mutual giving and receiving of concern and care. However, *love* also comes with a sense of responsibility towards the other. From the perspective of Aquinas (1948), we might say that our consistent willing and choosing the good of another is proof of our *love* for them. Therefore, for the remainder of this paper, we will define *love* as both a felt experience and an act of the will. *Love* is a strong affection towards another as well as the consistent choice made to achieve their good, even at the expense of our own.

In more contemporary philosophical analyses of love, some authors have identified core factors that influence the success or failure of a loving relationship. De Botton (2023), for example, identified several *anchors* or fundamental components that underpin a robust loving relationship, including non‐defensiveness and vulnerability. He defines vulnerability as the disclosing of one's true self, including perceived inadequacies or insecurities, to those around them. Vulnerability is not a weakness, but rather an act of courage that enhances our capacity to meaningfully connect with others (Brown 2015). It is of particular relevance to stuttering, given that openly stuttering and revealing oneself as a person who stutters is an act of vulnerability (Constantino 2019). Although de Botton (2023) is referring specifically to romantic loving relationships, the authors argue that this anchor has applicability to a broader range of relationships, including friendships, relationships with family members, relationships with colleagues, and relationships with new conversational partners. Given the evidence that suggests stuttering has the potential to enrich one's relationships with others, examination of this anchor and its applicability to stuttering might illuminate what it is about stuttering that can enhance these relationships.

## 4 Vulnerability

De Botton (2023) argues that someone who is good at love is good at being vulnerable. Revealing one's true self is no easy feat, and we sometimes lie or pretend to be someone else in order to maintain a love that we rely on (de Botton 2023). Examination of empirical research reveals that the concealment of disfluencies and avoidance of speaking situations are common aspects of the lived experience of stuttering (Connery et al. 2019). Concealment of the observable moments of stuttering is a protective and reactive strategy that is used as a result of the social stigma and discrimination that individuals who stutter have to endure (Constantino et al. 2017; Gerlach‐Houck et al. 2023). There are numerous forms of concealment, including situational avoidance (e.g., choosing not to verbally participate in class) or linguistic concealment (e.g., switching words) (Gerlach‐Houck et al. 2023). Concealment often commences in early childhood, which is evidence of the significant impact that societal norms and expectations have on a child (Gerlach‐Houck et al. 2023). Children demonstrate preferences for playing with peers who do not stutter from as early as 4 years old, and children who stutter are at a higher risk of experiencing peer rejection and bullying than children who do not stutter (Blood and Blood 2004, 2007; Davis et al. 2002; Ezrati‐Vinacour et al. 2001). Further, stuttering is associated with reduced earnings and other workplace challenges in adulthood (Connery et al. 2019; Gerlach et al. 2018). It is therefore unsurprising that many children and adults respond with concealment of their disfluencies and avoid disclosing their true selves to those around them. Concealment, in other words, is a reasonable response to stuttering in a hostile world (Constantino et al. 2017). Empirical research has found that efforts to conceal stuttering and the reduced disclosure of stuttering are, however, linked to a poorer quality of life and increased psychological distress for adults who stutter (Boyle et al. 2018; Gerlach et al. 2021). When people who stutter have fluency as a goal when speaking, they have significantly more repetitive negative thoughts than when they have the goal of stuttering openly (Tichenor et al. 2023). Such efforts to protect oneself from the stigma linked to speaking differently, therefore, have significant long‐term consequences for the individual.

De Botton (2023) argues that our perceived inadequacies do not disappear because we have hidden them. Rather, we make it challenging for people to truly know us, and consequently, others refrain from showing their true selves. For it is only by the mutual disclosure of true selves or the reciprocated ‘*nakedness’*, that a constructive relationship can be formed (Constantino 2019; Gerlach et al. 2021). Openly stuttering leads to an individual being momentarily defenceless or ‘*stripped naked*’ and is an indication to the listener that the conversational space is a safe one (Constantino 2019, 220). By lowering one's guard with stuttering, an individual can trigger the listener to lower theirs, and to reveal their true self (Constantino 2019). A byproduct of both the speaker and the listener being mutually vulnerable is *intimacy* (the feeling of closeness or connection with another person at an emotional, intellectual or sexual level) (Constantino 2019). Intimacy is a fundamental component of all healthy relationships and fosters an individual's mental and physical health (Khalifian and Barry 2020; Pietromonaco et al. 2013; Uchino 2009). Openly stuttering, therefore, has the capacity to facilitate the mutual disclosure of true selves, resulting in an intimate connection between two people, something that can potentially lead to a loving relationship. It is only after we know each other that we can love each other (Constantino 2025).

Williams (2023) argues that disclosing that one stutters to a listener, for example by openly stuttering, without knowing how the listener will react, is a form of risk‐taking. The individual is choosing to place themselves in a vulnerable position, typically not knowing what the consequences will be. Such risk‐taking may cause discomfort initially, however, it has the potential to lead to benefits for both the individual who stutters and the listener. Empirical research has found that listeners are more likely to perceive speakers who self‐disclose their stuttering (with a self‐disclosure statement) as more confident, friendly, and outgoing than speakers who do not self‐disclose (Byrd, McGill et al. 2017). In addition, disclosure to a listener in an informative and neutral manner (i.e., not apologetic) results in more positive observer perceptions of a person who stutters (i.e., they are perceived as more friendly and more confident than speakers who disclose in an apologetic manner; Byrd, Croft et al. 2017). This research on disclosure provides evidence that revealing oneself as a person who stutters directly influences a listener's perceptions of the speaker. Importantly, the value of self‐disclosure is not exclusive to listeners’ perceptions but also leads to benefits for the person who stutters. In a qualitative study by Young et al. (2022), adults who stutter reported that self‐disclosure increased listener engagement, and they described listeners as being more patient, enthusiastic, less reactive to stuttering moments, and more attentive to the content of their message following self‐disclosure. Further, participants in this study reported that having open conversations with friends and family about their experience of stuttering (sometimes for the first time) led to increased feelings of authenticity and emotional intimacy (Young et al. 2022). The research on self‐disclosure, an act of vulnerability, highlights the positive impact that this strategy has for both conversational partners and the social connection that they are co‐constructing.

Simply being a person who stutters does not automatically create vulnerability. The speaker must cooperate with the stutter, allowing it to happen, for the moment of vulnerability to be created. Plexico et al. suggested that there are two ways a speaker might react to their stuttering: by attempting to avoid it to protect themselves and others (Plexico et al. 2009a) or by approaching the moment in an open, agentic way (Plexico et al. 2009b). Constantino (2023) called the former avoidant response to stuttering a *stutterphobic* response because it represents a dislike and rejection of stuttering. He called the latter approach a response to stuttering, a *stutterphilic* response because it represents and embraces an attraction to stuttering. Stutterphobic responses, in so far as they obscure a person's authentic self, interrupt and fight against vulnerability. This is why many people who are still struggling with their stuttering can find it so isolating and disruptive to relationships (Corcoran and Stewart 1998). It is only when the moment of stuttering is allowed to happen that the speaker introduces vulnerability to the conversation, inviting their listener to reciprocate it. This mutual vulnerability is what allows us to know each other and, ultimately, to love each other (Constantino 2025). Stuttering support groups represent safe and non‐judgemental environments in which people who stutter can gather as a community, exchange their thoughts, feelings and experiences, and where mutual vulnerability can be facilitated (Bloye et al. 2023). Research has confirmed that adults who stutter initiate participation in such groups primarily to meet other people who stutter, and they maintain attendance at these groups because of the relationships they have developed (Yaruss et al. 2002). Interacting with other group members who are experiencing similar feelings, emotions and attitudes in relation to their stuttering can create a special connection and mutual understanding between members (Medina et al. 2020). In Medina et al.'s (2020) qualitative study that explored the narratives of adults who stutter attending stuttering support groups, participants described how beneficial it was to stutter openly and to observe others stuttering openly. As one participant expressed: “*I love coming to these groups because I feel like I can stutter*” [P1] (Medina et al. 2020, 147). Research on stuttering support groups, therefore, alludes to their important role as contexts that facilitate mutual vulnerability with consequential relational benefits for their members.

That disabilities make one vulnerable is not unique to stuttering. In fact, disability scholars have often been critical of a kind of intimacy hoisted upon the disabled, often against their will (Quinn 2024). Mingus (2017) calls *forced intimacy* the experience of having to share personal information about oneself and allow others to see and help one in compromising ways to survive in an ableist society. This is vulnerability against one's will and is rightly critiqued as a way people are disabled over and on top of their impairments. Forced intimacy is a compulsory transaction: the person is forced to divulge intimate details about themself and their disability to navigate the world. However, that is not what we mean by intimacy here. We are not talking about access or transaction, but rather the opportunity for mutual intimacy. This is voluntary, not compulsory, intimacy. Mutual intimacy is not a transaction, nor can it be compelled. In a transaction, people are trading different things. In mutual intimacy, both people are joining together to get the same thing, communion (Constantino 2025). There is a joint creation of something that was not there before. In forced intimacy, no consent is given for the vulnerability (Mingus 2017). But here we are speaking of voluntary vulnerability. The voluntary nature implies consent. What we are discussing is much closer to another of Mingus's concepts. She calls *access intimacy* the feeling of ease that occurs with certain people who implicitly understand her access needs, a feeling of safety that allows for vulnerability (Mingus 2011). Mingus suggests that this access intimacy precedes vulnerability and is maybe even a precondition for vulnerability. We are suggesting something slightly different, that the person who stutters can unilaterally make themselves vulnerable as an invitation to the other for mutual vulnerability. It is only when vulnerability is mutual that we can have intimacy. Mingus places intimacy before vulnerability, while we place vulnerability before intimacy; nonetheless, the concepts are analogous.

To summarise, vulnerability is a key component that underpins all robust loving relationships. Stuttering openly, an act of vulnerability, has the potential to enhance the relationships that people who stutter experience. Concealment of disfluencies, and ultimately one's true self, is a protective strategy that many people who stutter implement to shield themselves from societal stigma and discrimination against stuttering. The use of this strategy, however, has negative implications for individuals who stutter in terms of quality of life and psychological well‐being. Increasing evidence for the benefits of self‐disclosure and support group attendance highlights the importance of revealing one's true self (Boyle & Gabel, 2020; Gerlach et al., 2019; Healey et al., 2007; Herring et al., 2022; Werle & Byrd, 2022; Young et al., 2022). These activities have the potential to facilitate mutual vulnerability and enhance relationships with conversational partners, family, friends, and fellow stutterers. In essence, revealing oneself as a person who stutters affords the opportunity to truly know and love one another.

## 5 Clinical Implications

Based on the literature reviewed above, key clinical recommendations to bolster clients’ enactment of vulnerability and enhancement of their relationship experiences will now be outlined. Such recommendations have the potential to positively influence clinical practice by modifying SLTs’ perspectives on stuttering and their choice of therapies, thus enhancing outcomes for their clients.

1. Encourage clients to self‐disclose

As discussed, there is a growing body of evidence highlighting the benefits of self‐disclosure for both speakers who stutter and their listeners. SLTs should encourage and provide practice opportunities for children and adults who stutter to self‐disclose their stuttering to people in their environment (in school, workplace settings, etc.), either by stuttering openly or by using a disclosure statement. Disclosing their stuttering in a neutral and informative manner is critical to maintaining the positive effects of self‐disclosure (Byrd, Croft et al. 2017). Byrd, McGill et al. (2017) caution that clinicians should encourage rather than force self‐disclosure, as some people in the client's context may find it difficult to disclose to. It is therefore important that clients are adequately supported, as some may require additional assistance with managing negative listener reactions. The use of virtual reality as a tool to expose clients to anxiety‐inducing stimuli, such as self‐disclosure, is encouraged (Chard and van Zalk 2022).

2. Consider the use of therapies that aim for authenticity and the reduction of concealment

Recent research highlights the unhelpful and potentially harmful nature of fluency‐shaping therapies or approaches that promote stutter‐free speech (Gerlach‐Houck et al. 2023). Such therapies promote the concealment of stuttering and the individual's true identity. Research has confirmed that clinicians overestimate the benefits of healthcare interventions and underestimate the harms of interventions, which has the potential to lead to suboptimal clinical decision‐making (Hoffman and Del Mar, 2017). Providing a client with information on the range of intervention options in order for them to make an informed decision about their intervention path is an important role of the SLT. SLTs should be mindful that the facilitation of a supportive decision‐making process in collaboration with the client is an important measure of intervention success (McCurtin et al. 2024). SLTs are advised to offer therapies with contrasting underpinning philosophies to those of fluency‐shaping therapies. One such therapy, Avoidance Reduction Therapy for Stuttering (ARTS), facilitates the confrontation of the fear of stuttering, thus aligning with the revelation of one's true self as an individual who stutters (Sisskin and Goldstein 2022). One of ARTS' five therapy outcomes, for example, centres on finding joy in communication by increasing engagement and connection with others, and by sharing one's personal stuttering experiences with others. SLTs should initiate training in such therapies that support an individual's revealing of their authentic self and present them as therapeutic options for children and adults.

3. Encourage support group participation

As discussed, stuttering support groups afford a range of benefits to people who stutter, including the facilitation of mutual vulnerability with consequential relational benefits for participants. SLTs should therefore encourage their clients’ engagement with such groups to complement speech and language therapy goals.

4. Encourage parents’ modelling of vulnerability

Parents can be encouraged to model being vulnerable with their children to create a safe place for their child to reveal their authentic self (e.g., for the child to stutter openly or to talk about their thoughts and feelings relating to stuttering). Parents can, for example, apologise for mistakes made, talk about struggles or failures they have themselves experienced and the important role that these experiences have played in growing as a person. Parents can also show a willingness to be in solidarity with their children who stutter. It can be fruitful for parents to ask their children to teach them to stutter as they do and, with their children's permission, to use this stuttering with others. This positions their child as the expert, provides many opportunities for debriefing and discussion, and develops empathy. Perhaps most importantly, it allows parents to model vulnerability by doing something difficult.

5. Create a safe clinical space to facilitate mutual vulnerability

An effective therapeutic relationship requires similar conditions to those that are necessary for a loving relationship to be established. SLTs are advised to create a safe and non‐judgemental space for their clients to ensure they feel comfortable with being vulnerable in the clinical setting. Carl Rogers has referred to this orientation to the client as *unconditional positive regard* (Rogers 1957). Be mindful that this may be the first time that they are revealing themselves as a person who stutters, and it is therefore essential that the SLT responds in a supportive manner. Take time to establish a trusting relationship with them by actively listening to their story and responding with empathy (Byrne and Connery, 2023). Research in the discipline of psychotherapy has traditionally viewed vulnerability as being a task for which the client has sole responsibility (e.g., disclosing their thoughts, feelings and fears to the therapist; Powers 2017). However, more recent research has reconceptualised vulnerability as a relational process in which both the client and the therapist are open to being vulnerable throughout the therapeutic relationship (Leroux et al. 2007; Powers 2017). SLTs should therefore be mindful of the role of the expression of their own vulnerabilities in enhancing the therapeutic alliance. Examples include allowing the client to see them in a moment of emotional expression (e.g., crying when listening to their story about their struggle with stuttering) or revealing that they are uncertain if the current therapy is best suited to the client due to poor therapy outcomes. This aligns with Carl Rogers’ (1957) identification of counsellor *congruence* or authenticity as one of the core conditions for enabling a client's psychological change to occur. Rogers (1957) explains this as the counsellor willing to be the person that they are, to enter into a relationship with the client as an equal rather than an expert, to be aware of the thoughts and feelings that arise in them, and to communicate these, as appropriate, with the client. SLTs demonstrating their own vulnerability provides a model to the client that has the capacity to prompt them to be open and authentic about their stuttering. Importantly, SLTs are advised to consider appropriate ethical guidelines around therapist self‐disclosure and scope of practice, to ensure their behaviour aligns with what is expected of them as registered therapists (e.g., code of professional ethics provided by professional health regulators (e.g., CORU in Ireland; CAA in the USA) and professional bodies (e.g., IASLT in Ireland; ASHA in the USA).

6. Explore the concept of *stuttering gain*

Stuttering gain acknowledges the valuable experiences that an individual has because of their stuttering, whilst also recognising the challenges of stuttering that co‐exist (Constantino 2016; Constantino et al. 2022). SLTs should explore stuttering gains with their clients and support them in identifying any positive aspects of their experiences with stuttering. This may help them to view their stuttering as multi‐dimensional, offering both benefit and adversity, similar to most other human qualities (Constantino et al. 2022).

7. Extend knowledge on vulnerability to other contexts

The relationship between a supervisor and a supervisee, and their mutual vulnerability, is at the core of an effective supervision process (Bradley et al. 2019; Duffey et al. 2016). SLTs are advised to model and promote mutual vulnerability in their clinical supervision with SLT students or with colleagues. SLTs can, for example, model vulnerability by sharing past experiences of clients that they struggled to work with or discuss therapies that they regret implementing due to poor therapy outcomes. By sharing these experiences and stepping out of their comfort zone, it will give permission to the supervisee to do the same, thus enhancing the supervisory relationship.

## 6 Recommendations for Future Research

Exploration of the advantageous by‐products of stuttering is required to comprehensively understand the lived experience of stuttering for all individuals. Most studies examine the negative impact of stuttering and how it interferes with relationships and an individual's quality of life. There is a dearth of research examining how stuttering might deepen relationships or provide opportunities for new, robust relationships. Qualitative research exploring the role of stuttering in introducing vulnerability into our conversations, and its capacity to enable our knowing and loving of one another, is recommended. Further, future research on topics such as the lived experience of stuttering needs to broaden the diversity of participants recruited and include those who have not sought external help for their stuttering. The majority of participants in studies on this topic are recruited through speech and language therapy clinics or self‐help/support groups, and findings may therefore not be generalisable to all people who stutter, in particular, those who have chosen not to seek support (Connery et al. 2019). The life experiences of such participants need increased attention in the literature. It may be that the experiences of such individuals are less adverse, and some of the more advantageous by‐products of stuttering may be illuminated by their inclusion in research.

## 7 Conclusion

Although the literature highlights the mostly negative impact stuttering has on an individual's life, more recent evidence alludes to alternative and more positive experiences for some adults who stutter. Aspects of these alternative experiences include enhanced interpersonal relationships and increased sensitivity to others. Exploration of such advantageous by‐products of stuttering is lacking in the field, and, to comprehensively understand the lived experience of stuttering for all individuals, such investigation is required. One explanation for the enhanced relationships lies in the nature of openly stuttering or facilitating the sharing of one's true self with another, thus opening up the opportunity for the other person to reveal their true self (i.e., mutual vulnerability). It is only in this communion that love is possible. Examination of early and more contemporary literature on loving relationships highlights the multiple definitions and types of love used over centuries and across disciplines. More contemporary conceptualisations of love indicate the importance of vulnerability as a fundamental component that underpins a robust loving relationship. Concealment of disfluencies and avoidance of disclosing one's true self are common features of the lives of children and adults who stutter. These efforts to shield oneself from the stigma linked to speaking differently have significant long‐term implications for the individual, such as reduced quality of life. Conversely, stuttering openly can facilitate the mutual disclosure of true selves, resulting in an intimate connection between two people. In this way, stuttering can act as a precursor to love. SLTs are advised to consider the range of clinical recommendations discussed in this paper to support their clients’ enactment of vulnerability and enhancement of their relationship experiences. These include supporting clients to self‐disclose, offering therapies that aim for authenticity and the reduction of concealment, and encouraging clients’ engagement with stuttering support groups. Future research should further explore the advantageous by‐products of stuttering and investigate the role that stuttering plays in enabling and enhancing relationships. To conclude, this paper presents a novel conversation on the capacity for stuttering to heighten a person's cultivation of robust relationships. It contributes to the current dialogue that challenges traditional deficit‐based perspectives of stuttering and aligns with the growing movement of neurodiversity and social model approaches in speech and language therapy. Such dialogue continues to shift perspectives in stuttering research whilst also influencing clinical practices used by SLTs to enhance the lives of those who stutter.

## Conflicts of Interest

The authors have no conflict of interest to report.

## Data Availability

Data sharing is not applicable to this article as no datasets were generated or analysed during the current study.
